# Environmental enteropathy and its association with water sanitation and hygiene in slum areas of Jimma Town Ethiopia

**DOI:** 10.1371/journal.pone.0286866

**Published:** 2023-06-23

**Authors:** Rediet Regassa, Dessalegn Tamiru, Markos Duguma, Tefera Belachew

**Affiliations:** 1 Nutrition and Dietetics Department, Faculty of Public Health, Institute of Health, Jimma University, Jimma, Ethiopia; 2 Jimma University Laboratory of Drug Quality (JuLaDQ) and School of Pharmacy, Jimma, Ethiopia; Cranfield University, UNITED KINGDOM

## Abstract

**Background:**

Environmental Enteropathy is an inflammatory condition of the gut that leads to intestinal barrier dysfunction. It is a common problem in resource-limited countries and results from exposure to larger quantities of fecal bacteria to poor personal hygiene and environmental sanitation. Due to poor intestinal permeability, there is a problem with absorption of nutrients, which in turn leads to growth faltering, poor cognitive development, and oral-vaccine failure. The aim of this study was to identify the children with an elevated lactulose to mannitol ratio (indicative of possible environmental enteropathy) and its association with water sanitation and hygiene in slum areas of Jimma Town so as to mitigate the problem of malnutrition in under-five children.

**Methods:**

A community-based cross-sectional study was carried out from January to April 2021. A Lactulose mannitol test was performed to determine the prevalence of elevated lactulose to mannitol ratio (possibly environmental enteropathy) in children aged 12 to 59 months. A pretested questionnaire was used to collect data on water sanitation and hygiene (WASH) indicators and sociodemographic characteristics. A multivariable logistic regression analysis was used to isolate independent predictors for possible environmental enteropathy. All tests were two-sided and statistical significance was declared at P<0.05.

**Results:**

The results of this study showed that 19.3% (95%CI: 14.8–23.7) of children had an increased lactulose to mannitol ratio (>0.15). On multivariable logistic regression analysis, the variables drinking water from unimproved water sources (AOR 3.741; 95%CI: 0.914–15.310,p = 0.048), unsafe coverage of water storage (AOR 0.363; 95%CI: 0.169–0.777, P = 0.009), public latrine utilization (AOR 0.139 95%CI: 0.024–0.816, P = 0.029),and hand washing less than 3 critical time of hand washing practices (AOR 4.369;95%CI: 1.411–13.524,P = 0.011) were significantly associated with an increased in lactulose mannitol ratio (possible indicative of intestinal permeability/environmental enteropathy).

**Conclusion:**

This study showed that one fifth of under-five children in Jimma Town had an elevated lactulose to mannitol ratio (possibly environmental enteropathy). The WASH sectors and other governmental organizations should give emphasis to areas with poor water sanitation and hygiene to mitigate the problem of environmental enteropathy and related consequences like growth faltering, poor cognitive development, and oral-vaccine failure in the study area.

## Introduction

### Environmental enteropathy

Environmental enteropathy, also known as environmental enteric dysfunction, is a condition of intestinal structure and function that mostly affects children living in resource-constrained environments [1]. Poor hygiene and unsanitary environmental conditions are hypothesized to cause environmental enteropathy, as they lead to recurring enteric infections, which cause intestinal inflammation, impaired absorptive capacity, disturbed barrier function, and, eventually, leaky gut syndrome [2]. Chronic exposure to fecal pathogens is thought to promote inflammation in the small bowel, which leads to structural abnormalities and, eventually, functional changes that lead to intestinal permeability (environmental enteropathy). These functional alterations, which include gut barrier disruption, carbohydrate malabsorption, and chronic inflammation, are thought to play a role in reduced gut immune function and oral vaccine failure, as well as growth failure and decreased infant development [3].

### Environmental enteropathy and child growth

Changes in intestinal morphology and underlying chronic inflammation affect nutrient intake and thus impair normal linear growth in children [4]. The number of stunted children worldwide was 150.8 million, and the number of wasting children was 50.5 million. The vast majority (95%) of them were in Asia and Africa, including Ethiopia [5]. Ethiopia has a high prevalence of stunting, with rates ranging from 49% in Tigre to 14% in Addis Ababa, and wasting varies across the country, with rates as high as 21% in Afar [5]. Multiple and repeated intestinal infections are more common (with and without overt diarrheal illnesses) in young children living in impoverished areas lacking adequate sanitation or clean water [6]. There is a cyclical relationship between poor nutrition and susceptibility to infection in determining nutritional status. Poor nutrition, increased susceptibility to infectious diseases, leading to immunological dysfunction and metabolic responses that further alter nutritional status and, wherever possible, related physiological mechanisms [7].

Unsanitary and unhygienic conditions, inadequate water sanitation and hygiene conditions, animals in a child’s sleeping area, poor caregiver hand hygiene, putting dirt objects in their mouths, unsafe disposal of child feces, and fecal ingestion were the main causes of environmental enteric dysfunction [8]. It was the main contributing factor for EED and stunting in low and middle income countries [9]. There is also a significant association between fecal markers of EED and stunted growth in young children [10].

Repeated episodes of diarrhea, infections transmitted by helminthes in the soil, and environmental enteropathy have all been proposed as the key routes linking inadequate water sanitation and hygiene (WASH) and stunting [11]. WASH intervention delivered with sanitation education, hand washing with soap, availability of sanitary facilities at HHs, a clean environment at HHs, and separate housing of animals showed a gain in height in children age 6–36 months [11].

### Lactulose mannitol absorption test

The best diagnosis for EED was a small intestinal biopsy. The lactulose-mannitol test was the most non-invasive proxy marker which is a widely used alternative method in which lactulose and mannitol are digested and urine is collected over the next several hours. Interrupted cellular binding allows lactulose, a disaccharide, and mannitol, a monosaccharide, to be passively absorbed, providing a measure of the integrity of the intestinal epithelium [9]. Lactulose is too large to cross normal mucosa unless intestinal permeability across the tight junctions between epithelial cells is excessive. Mannitol, on the other hand, is handled like other monosaccharaides that are taken up in proportion to small-bowel absorptive capacity. Absorbed lactulose and mannitol are then filtered at the glomerulus and are not reabsorbed by the kidney. Therefore, their relative concentrations in urine and the absolute amount of mannitol, respectively, measure intestinal permeability and small-bowel absorptive capacity [12].

The urinary lactulose to mannitol ratio (L:M) indicates the extent of epithelial disruption in the small intestine and persistent poor gut health [9]. Thus, urine mannitol is an indicator of absorbability, while the presence of lactulose in urine indicates impaired barrier function [13].

### Water sanitation and hygiene conditions in Ethiopia

Access to clean water, basic sanitation, and hygiene are the main factors for nutritional wellbeing. In 2019, 67 percent of Ethiopian households used improved water sources, a 2% increase from 2016. Even though Ethiopia has seen a dramatic decline in open defecation, only 18% of households used an improved toilet facility in 2019. In Ethiopia, the availability of basic hygiene facilities is still relatively limited. According to the 2016 EDHS [14], only 13% of households have hand-washing facilities with soap and water.

In all, 20% of Ethiopian families have access to modern toilets (42% of urban households and 10% of rural households). Twenty-seven percent of families lack access to a toilet (35 percent in rural areas and 10 percent in urban areas). Over 56% of rural homes have unimproved sanitary facilities [5].

Safe drinking water, proper sanitation, and hygiene can prevent under nutrition and stunting in children by preventing the development of environmental enteropathy and diarrheal disease. Reductions in diarrheal disease alone through safe WASH can prevent long-term morbidity and at least 860,000 child deaths a year caused by under nutrition [15].

### Wash factors, environmental enteropathy, and malnutrition hypothetical pathways

Water, sanitation, and hygiene (WASH) have traditionally been linked to acute gastrointestinal infections. Recently, it has been hypothesized that an important pathway through which inadequate WASH access impacts the burden of disease is via chronic inflammation in EED [3]. Intestinal infiltrative/mucosal damaging intestinal pathogens have been linked to intestinal inflammation and infant growth retardation [16].

Repeated episodes of diarrhea, infections transmitted by helminthes in the soil, and environmental enteropathy have all been proposed as the key routes linking inadequate water sanitation and hygiene (WASH) and stunting. The socio-economic environment is also responsible for the additional indirect linkages between poor WASH conditions and nutritional status, such as access to and affordability of WASH services, the distance from a family to a water source, education, and poverty [17]

Poor sanitation and hygiene cause stunting not only through diarrhea but also through the subclinical condition, environmental enteropathy (EE). Exposure to larger quantities of fecal bacteria due to poor sanitation and hygiene is the cause of this enteropathy, now termed "EE." The primary causal pathway from poor sanitation and hygiene to stunting is EE, not diarrhea. The EE is characterized pathologically by the manner of approach to villous blunting; it is likely to result in a reduction of the surface area of the mature absorptive intestinal epithelial cells. It is conceivable that the ones adjustments to the gut form ought to limit nutrient uptake; truly expatriates with enteropathy have been positioned to have reduced intestinal function at the side of decreased absorption of carbohydrates, fat, and diet B12 [18].

**Fig 1:**
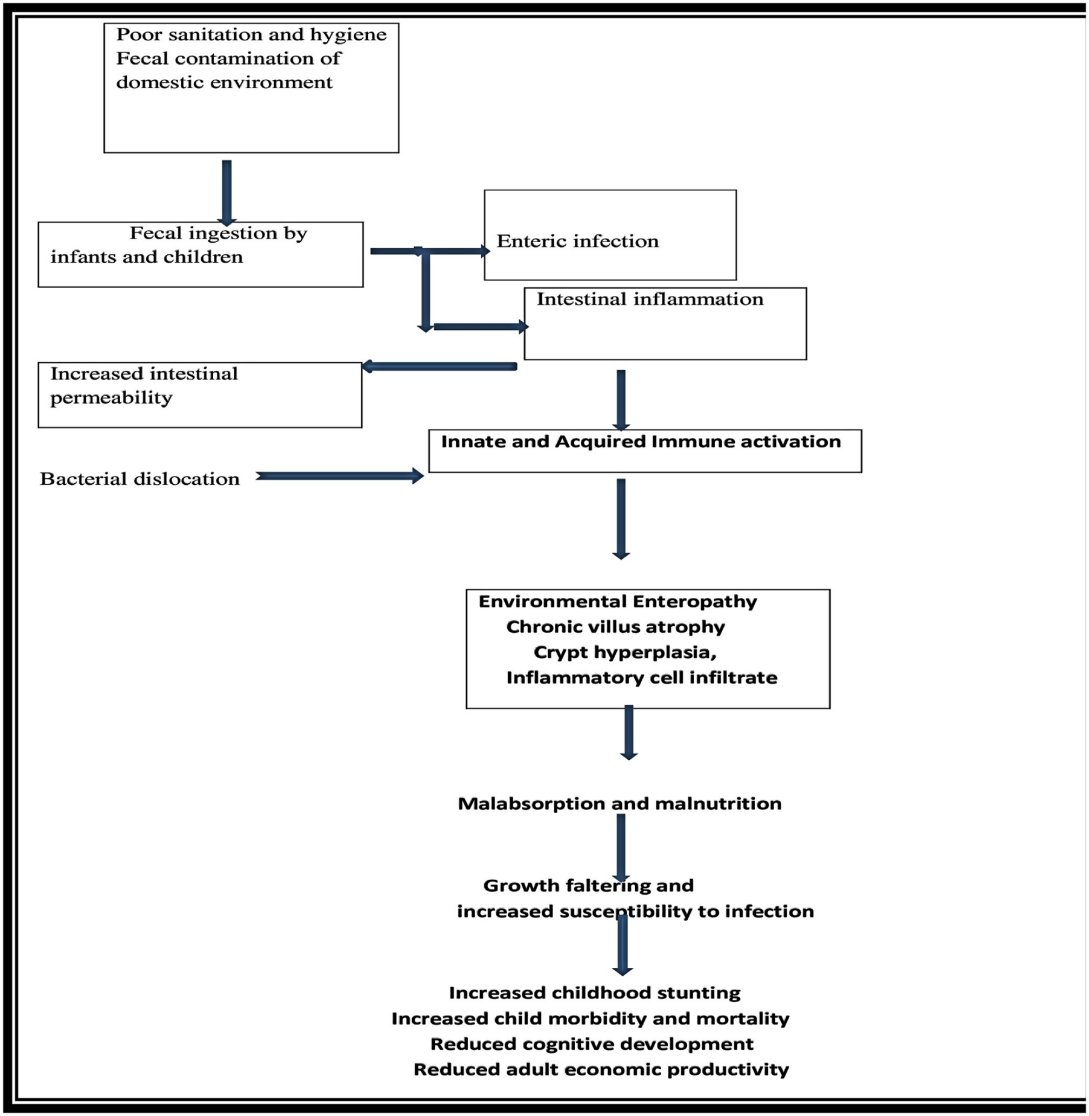
Hypothesized pathway of water, sanitation, and hygiene factors, environmental enteropathy, and malnutrition.

Poor household environments that likely predispose to enteric infections, Ingestion of microorganisms related to poor water sanitation and hygiene is postulated to result in morphological changes, leading to intestinal epithelial damage, increased permeability, and microbial translocation into the lamina propria. This invasion activates an inflow of inflammatory cells to the gut and ends in local and systematic inflammation, resulting in the reallocation of resources normally directed toward child growth and development and the disruption of hormonal pathways that regulate growth plate activity in long bones. Chronic inflammation and reduced intestinal nutrient absorption are also hypothesized to affect brain development, inducing lasting negative effects on cognition, educational achievement, and linear growth [19] (Fig 1).

Due to environmental enteropathy, the impact of the absence of water sanitation and hygiene (WASH) on the health burden of malnutrition may be significantly underestimated [15]. Despite adopting optimal dietary habits, children are still in a malnutrition cycle, leading to stunting. Stunting is the result of nutritional problems and the cumulative effects of recurrent or persistent infections, causing a continuous failure to thrive with age [3].

Though different studies were conducted in relation to environmental enteropathy and stunting and its association with a problem in water, sanitation, and hygiene in different countries, there is limited study that documented environmental enteropathy and its association with water, sanitation, and hygiene in the study area. This study set out to document the prevalence of a higher lactulose to mannitol ratio (possibly indicative of environmental enteropathy) and its association with water sanitation and hygiene factors to mitigate the problem of malnutrition in under five year-olds due to the consequences of environmental enteropathy.

#### Objectives

The aim of this study was to identify children with an elevated lactulose to mannitol ratio, indicative of possible environmental enteropathy and its association with water sanitation and hygiene problems, in order to deal with the problem of malnutrition and other consequences such as oral vaccine failure and impaired cognitive development in under-five children in the study area.

## Methods

The study was conducted from January to April 2021 in slum areas of Jimma Town. All the authors were involved in recruiting participants and data collection in the study areas. Jimma town is located in the south-west part of Ethiopia, at a distance of 357 kilometers from the capital city, Addis Ababa. The town has 13 urban small administrative areas and 4 rural small administrative units. In 2021/2022, the population of the town was 207,573. Over the course of the year, the temperature typically varies from 48°F to 83°F and is rarely below 42°F or above 88°F. The wet season is mostly cloudy; the dry season is partly cloudy. The town is bounded by Kersa woreda in the east, Manna woreda in the west, Mana and Seka woreda in the north, and Seka woreda in the south.

### Participation

Environmental enteric dysfunction in this study was measured using the lactulose-mannitol dual sugar absorption test. Although EED can only be diagnosed definitively through a small intestinal biopsy, the lactulose-mannitol test is the most commonly used, noninvasive proxy marker. In the test, mannitol recovery rates indicate absorptive capacity, lactulose recovery rates indicate permeability, and higher Lactulose to mannitol ratios (L: M) indicate greater intestinal abnormality, or EED [20].

Even though different studies used different cutoff points for EED, the majority of the study took 0.15 as the cutoff point [19]. A study done on Malawian children also used 0.15 as the cutoff value for the ratio. Accordingly, this study used this cutoff point for the ratio of dual sugars in determining the possibility of EED [9]. Health extension workers and lab technicians, who were able to communicate in Oromifa, were involved in data collection. Training was provided for data collectors and supervisors.

A day before administration of the lactulose mannitol solution Data collectors went to the homes of the children, gave general information about the test, took consent from caretakers, and gave instructions on preparation for the test.

A total of 300 children (140 males and 160 females), in the age group of 12 to 59 months (median age of 1 year), who were at risk for infection and, in turn, had intestinal dysfunction with no significant medical histories or gastrointestinal symptoms for 2 weeks before administration of the lactulose/mannitol solution, were involved in the test. After an overnight fast, the subjects then drank the test sugar solution containing 250 mg/ml of lactulose and 50 mg/ml of mannitol at a dose of 2 mL/kg up to a maximum of 20 ml. After 30 minutes, a liberal intake of water was permitted to increase urine flow. Food intake was allowed after the first 3 hours. The children were requested to empty their bladders before ingesting the test sugar solution, and a urine collection bag (McKesson Medical-Surgical lnc. MFR #4822, Richmond, VA) was placed and changed as needed for the 5-hour period [21]. To measure urine volume following voiding, 1–2 drops of chlorhexidine (20 mg/ml) were added, and samples were liquified and stored on ice to limit microbial growth. Urine aliquots were stored at -80°C before testing [22]. On completion of the study, samples were stored at −80°C in laboratory of Jimma University before being transported on dry ice to the laboratory of the Ethiopian Conformity Assessment Enterprise for analysis.

### Laboratory procedure

#### Sample preparation

0.5 gm of washed cation exchange resin was added to 2 mL of the thawed urine specimen, vortexed for 10 seconds, and centrifuged for 10 minutes at 3000 rpm. The supernatant layer was withdrawn and filtered through 0.2L to inject into the HPLC system for analysis [20].

### HPLC system and chromatographic conditions

**Fig 2:**
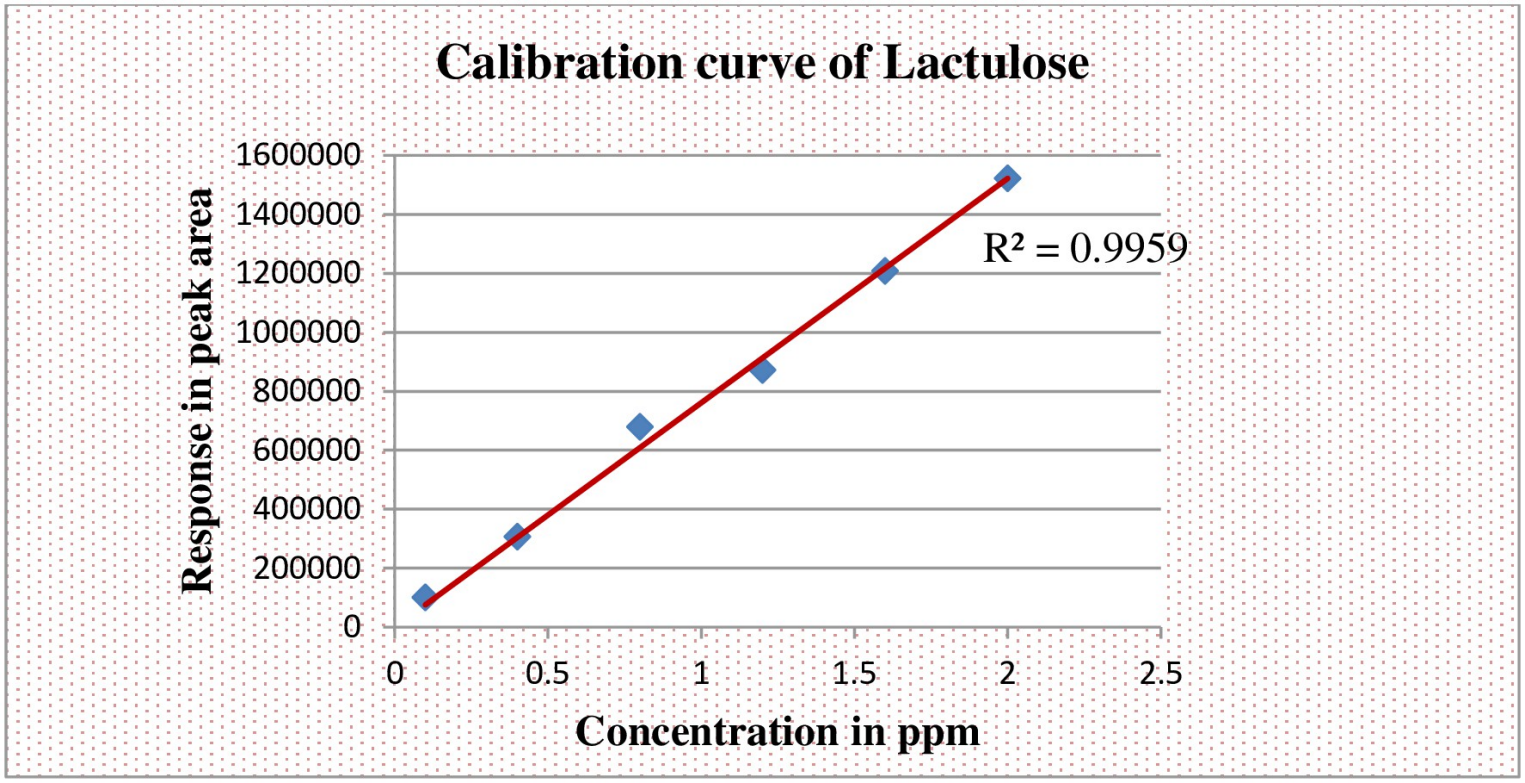
Standard curve when lactulose in aqueous solution is chromatographed.

**Fig 3:**
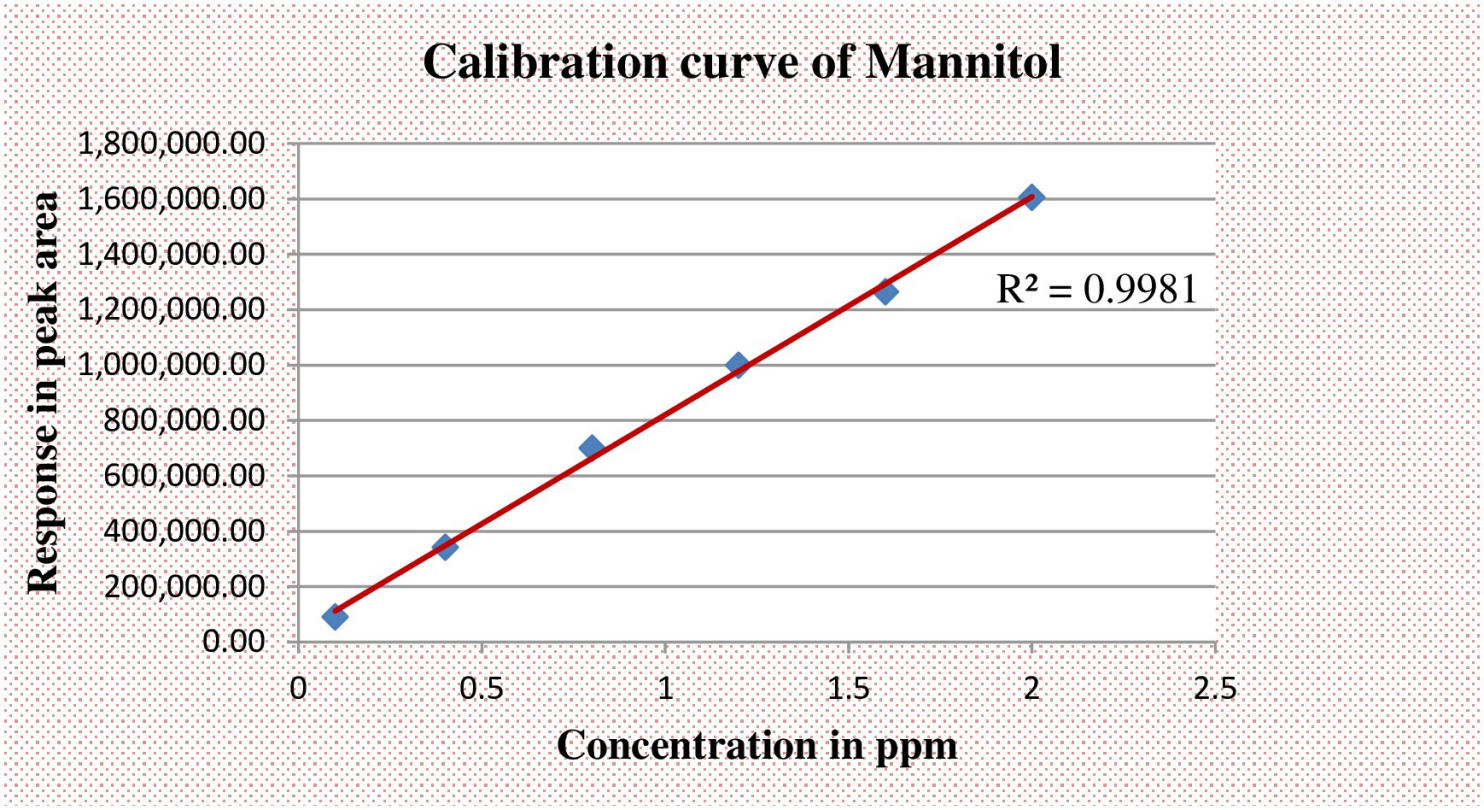
Standard curves when mannitol in aqueous solution is chromatographed.

The samples were analyzed using an Agilent 1260 Infinity Series HPLC system (Model SP 8810; Spectra Physics, San Jose, CsA) along with a carbohydrate column (4.6x150mm, 5μm) Zorbax, USA. The mobile phase was a mixture of acetonitrile and HPLC grade water (70/30%v/v). HPLC analysis was conducted at the column temperature, injection volume and flow rate of ambient temperature, 10.0 μl and 1.0 ml/min, respectively. The calibration curves were prepared by analyzing appropriate concentrations of each compound in distilled water and plotting the peak height obtained at 1.0 X l0 refractive index unit (full-scale) sensitivity of the detector. Urinary concentrations of sugar probes were calculated from the calibration curves by peak-area analysis (Figs 2 and 3).

### Variables of the study

The study was done to identify the outcome variable environmental enteropathy in children who were free from any medical history or GI symptoms for 2 weeks before the administration of lactulose mannitol solution for the diagnostic purpose. As the study was done in slum areas characterized by water sanitation and hygiene problems, indicators for water sanitation and hygiene problems were taken as predictors for the outcome variable.

### Data sources

A structured questionnaire was used to collect data on socio-demographic, health status, water, a sanitation and hygiene practices from care givers of under-five children through a face-to-face interview. A urine sample was collected after the administration of lactulose and mannitol solutions to determine intestinal permeability. During data collection, data collectors observed the hand washing facility, toilet area, and water storage practice using an observation checklist.

### Sample size determination

A total of 300 children in the age group of 12–59 months old were selected by using the single population proportion formula with an assumption of an estimated prevalence of (p) Environmental Enteropathy 50%, a 5% margin of error (d) and a 95% confidence level. A simple random sampling technique was used to select the study participants.

### Data analysis

Epi Data version 3.1 was used to enter and clean data, which was then transferred to SPSS version 26 for analysis. Urine analysis was performed by HPLC. Percent of excreted lactulose and mannitol in the urine was used to calculate the geometric mean of lactulose-to-mannitol ratio. Descriptive statistics such as frequency distribution and cross tabulation were done to summarize the study variables. Both bivariate and multivariable logistic regression was used to estimate crude and adjusted odds ratios with 95% confidence intervals. A multivariable backward step-wise regression model was fitted to adjust for potential confounding. To rule out excess variables and unstable estimates in the final model, only variables that reached p values ≤0.25 in the bi-variate analysis were included in the multivariable logistic regression analyses to control for associations among the independent variables. Model fitness was checked using the Hosmer-Lemeshaw test with a P>0.05 taken for fitness. The standard errors for regression coefficients <2.0, as a cut off value, showed that there was no multicollinearity among independent variables.

### Ethical statements

Approval of the study protocol was obtained from the Ethical Review Board of the University of Jimma, the College of Medicine and Health Sciences, the Institute of Public Health, and the Ethics Committee of the Oromia Regional Health Bureau. Before the study began, official permission was secured from each study district’s administrative as well as health offices. The purpose and importance of the study were explained to the participants. Written informed consent from a parent or guardian was obtained in accordance with the Declaration of Helsinki. The confidentiality of study participants was kept, and identification of study participants by name was avoided. All participants who were diagnosed with environmental enteropathy and anemia were linked to a nearby health facility for follow-up and management.

## Results

### Socio-economic and demographic characteristics with the lactulose to mannitol ratio

Based on the inclusion and exclusion criteria, 300 under-five children participated in this study. The number of children based on their age was 186 (62%), 114 (38%), for 1–3 and 4–5, respectively, with a mean age of 1.38 years, ranging from 1–5, and a standard deviation of ±0.5 years. The majority of study participants (53.3%) were females between the ages of 18 and 35 (88%) of their caregivers. A larger proportion of their parents (44.8%) and (37.7%) had no formal education. The majority of parents had a monthly income of < 3000 ETB (244) (81.3%), and the majority of children were from households with a family size of ≥5,182 (60.7%).

Of the study participants’ majority of children, 21% (= 39) fall within the age group of 1–3 years and had an elevated lactulose to mannitol ratio > 0.15, (COR 0.754; 95%CI: 0.411, 1.382, p = 0.361), indicative of possible EE. A nearly equal proportion of male and female children had elevated L: M > 0.15. The majority of children with an elevated Lactulose to Mannitol ratio fall within the youth age group of their caregivers >35, 25%(n = 9), (COR 1.463; 95% CI:0.647,3.307 p = 0.356),from family size of households ≥5, 21.4%(n = 39),(COR 0.704; 95%CI: 0.384,1.289 p = 0.255) and from caregivers who were married, 21.2%.(n = 39),(COR 0.814; 95CI:0.291,2.279 p = 0.695). A large proportion of children with indicative of environmental enteropathy were from mothers or caretakers with no education or a secondary educational level 20% (n = 11),(COR 1.116; 95% CI: 0.501,2.486 p = 0.866) and 21.8%(n = 24), respectively. Most of the children with an elevated lactulose to mannitol ratio of >0.15 were from house wives as mothers/ care givers 25.2%; n = 33), (COR 0.516; 95%CI: 0.289, 0.920; p = 0.025); and from households with a monthly income of ≥3000 Birr 30.4% (n = 17), (COR 2.158; 95%CI: 1.114, 4.180 p = 0.023) (Table 1).

**Table 1:** Socioeconomic and demographic characteristics associated with a lactulose to mannitol ratio (L: M), in slum areas of Jimma Town, Ethiopia (N = 300).

Socio demographic characteristics		L:M > 0.15		L:M ≤ 0.15		Crude Odds Ratio (95% CI)	P-value
		Percent	Number	Percent	Number		
**Child age**							
	1–3	21%	39	79%	147	.754(0.411,1.382)	.361
	4–5	16.7%	19	83.3%	95	1	
**Child sex**							
	Male	21.4%	30	78.6%	110	.778(.438, 1.381	.391
	Female	17.5%	28	82.5%	132	1	
**Age of care givers**							
	18–35	18.6%	49	81.4%	215	1.463(.647, 3.307	0.356
	>35	25%	9	75%	27	1	
**Household Family Size**							
	≥5	21.4%	39	78.6%	143	.704(.384, 1.289	.255
	<5	16.1%	19	83.9%	99	1	
**Maternal Marital Status**							
	Married	21.1%	39	76.9%	146	.814(.291, 2.279)	.695
	Divorced	16.1%	14	83.9%	73	1.134(.369,3.486)	.827
	Widowed	17.9%	5	82.1%	23	1	
**Maternal Education**							
	No Education	20%	11	80%	44	1.116(.501, 2.486)	.866
	Primary Education	17.2%	23	82.8%	111	1.347(.712, 2.548)	.360
	Secondary	21.8%	24	76.2%	86	1	
**Spouse’s level of Education**							
	No Education	19.5%	22	80.5%	91	.827(.092, 7.443)	.866
	Primary Education	10.5%	8	89.5%	68	1.700(.176, 16.432)	.647
	Secondary	25.7%	27	74,3%	78	.578(.065, 5.169)	.578
	College/university	16.7%	1	83.3%	5	1	
**Mother’s Occupation**							
	House wife	25.2%	33	74.8%	98	.516(.289, .920)	.025
	Self/government employed	14.8%	25	85.2%	144	1	
**Spouse’s Occupation**							
	Self/Government Employed	18.8%	18	81.2%	78	.946(.510, 1.756)	.861
	Unemployed	19.6%	40	80.4%	164	1	
**Households Average Monthly Income (ETB)**							
	<3000 ETB	16.8%	41	83.2%	203	2.158(1.114, 4.180)	.023
	≥ 3000 ETB	30.4%	17	69.6%	39	1	

### Lactulose-mannitol test

This study was aimed at determining the prevalence of children with an elevated lactulose to mannitol ratio and its association with water, sanitation, and hygiene. Even though the best diagnosis for EED was a small intestinal biopsy, the lactulose-mannitol test was the most non-invasive proxy marker. The result of the geometric mean SD of lactulose and mannitol concentration, % lactulose and mannitol excreted, and lactulose to mannitol ratio was calculated by using spss for mean comparison. The geometric mean of lactulose and mannitol concentrations was found to be 28.3757 ±28.70456 and 49.9819 ± 39.62776 respectively for males and 26.4418 ±21.13264 and 45.0755 ± 30.80682 respectively for females. The geometric mean SD of % lactulose excreted and % mannitol excreted was found to be 0.52± 0.11 and 4.101± 1.59 respectively, for males and 0.49 ± 0.91 and 3.86 ± 1.21 respectively, for females. The geometric mean SD of the lactulose to mannitol ratio (L: M) was found to be 0.17± 0.1and 0.15 ± 0.1 respectively for male and female. The lactulose to mannitol ratio of > 0.15 was taken as an indicator of possible environmental enteropathy **(**Table 2).

**Table 2:** Geometric mean of lactulose and mannitol concentration, percent (%) lactulose and mannitol excreted and lactulose to mannitol ratio (L: M) of under-five children in slum areas of Jimma Town, Ethiopia (N = 300).

Child sex	lactulose Conc.			Mannitol conc.			%lactulose excreted			% mannitol Excreted			Lactulose to mannitol ratio(LMR)		
	Number	Geo.mean	SD.	number	Geo.mean	SD.	Number	Geo.mean	SD.	number	Geo.mean	SD.	number	Geo. mean	SD.
**male**	140	28.3757	28.70456	140	49.9819	39.62776	140	0.52	0.117	140	4.101	1.59	140	0.17	0.1
**female**	160	26.4418	21.13264	160	45.0755	30.80682	160	0.49	0.91	160	3.86	1.21	160	0.15	0.1

### Prevalence of environmental enteropathy

**Fig 4:**
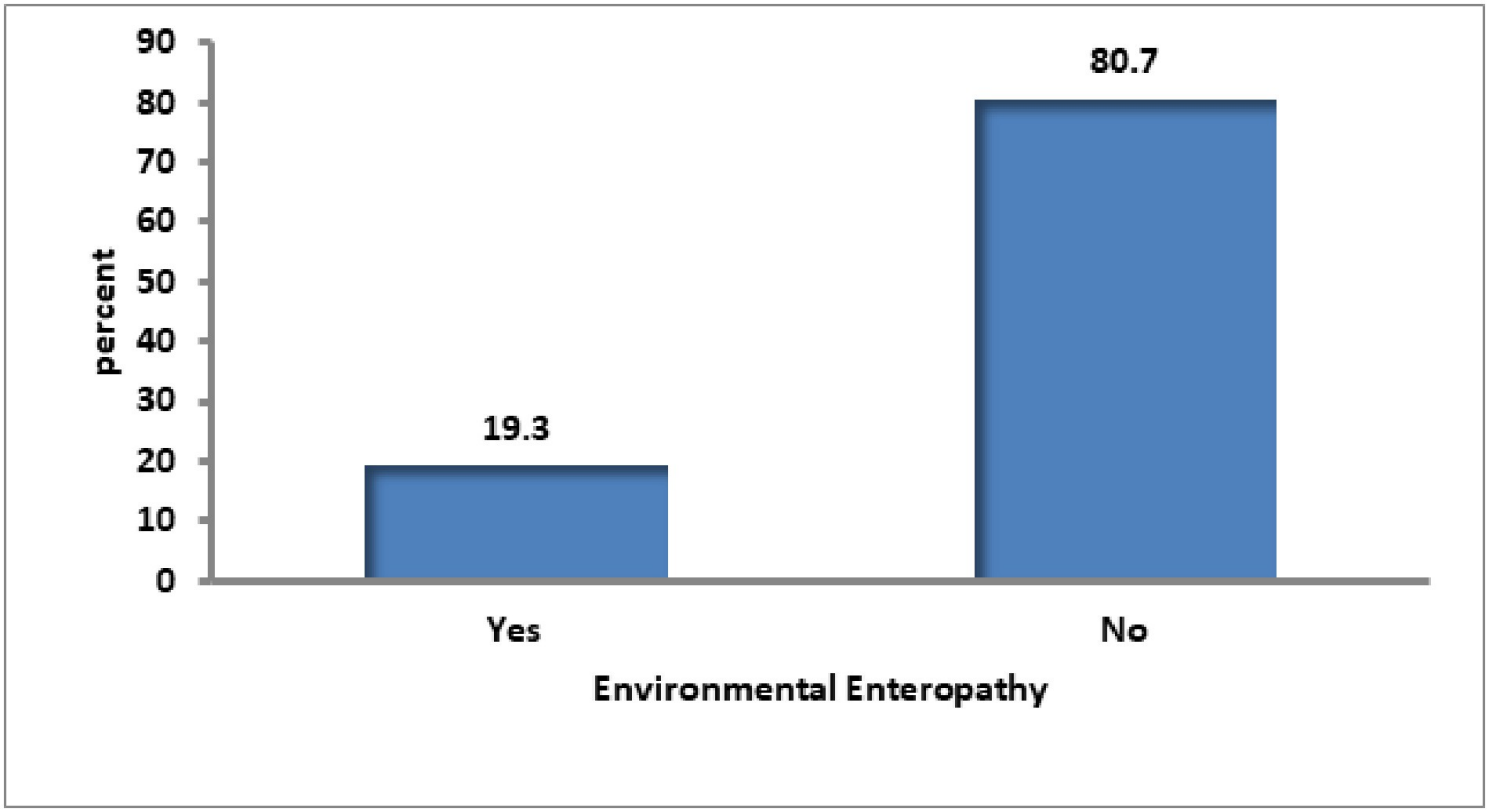
Prevalence of environmental enteropathy.

Environmental enteropathy was denoted as yes (1), indicating a lactulose to mannitol ratio of >0.15 or no (0), indicating a lactulose to mannitol ratio of ≤ 0.15. The findings of this study revealed that 58 (19.3%; 95% CI: 14.8–23.7) of the 300 under-five children in the study had an elevated lactulose to mannitol ratio, an indicator of possible environmental enteropathy (Fig 4).

### Water access and use associated with the lactulose to mannitol ratio

Thirty-three percent (33%) (n = 31) of under-five children were from households with unimproved water sources (COR: 3.262; CI: 1.808–5.888 P<0.001) had elevated lactulose to mannitol ratio (L:M), indicative of environmental enteropathy, Forty-nine (18.4%) of the children with an elevated lactulose to mannitol ratio > 0.15 were from households that did not practice water treatment at the point of use (COR: 0.599 (.262, 1.370, p = 0.225). Children from households with unsafe drinking water storage and coverage were 17.4% (n = 41) (COR 0.597; 95%CI: 0.312, 1.140, p = 0.118), and 31.5%(n = 17),) (COR 0.435; 95%CI: 0.224, 0.846, p = 0.014), respectively, with an elevated lactulose to mannitol ratio. Twenty-six (19.5%) of the children with an elevated lactulose to mannitol ratio (≥ 0.15; COR 0.975; 95%CI: 0.548, 1.736, p = 0.933) were from water sources within the reach of animals. In the bivariate analysis, children that drank water from unimproved water sources (P = 0.001) and from unsafe storage coverage (P = 0.014) were significantly associated with an elevated lactulose to mannitol ratio (L:M) (Table 3).

**Table 3:** Frequency distribution and association of lactulose to mannitol ratio (L: M) with water variables in the slum areas of Jimma Town, Ethiopia (N = 300).

Water source	L:M >0.15		L:M ≤ 0.15		Crude Odds Ratio (95% CI)	P-value
	Percent	Number	Percent	Number		
**Unimproved**	33%	31	86.9%	179	3.262(1.808–5.888)	P<0.001
**Improved**	13.1%	27	67%	63	1	
**House Hold water treatment**						
**Don’t use**	18.4%	49	81.6%	218	.599(.262, 1.370)	.225
**Use**	27.3%	9	72.7%	24	1	
**general water storage practice**						
**Unsafe**	17.4%	41	73.8	48	0.597(0.312,1.140)	0.118
**Safe**	26.2%	17	82.6	48	1	
**Water storage container coverage**						
**unsafe**	31.5%	17	68.5	37	0.435(0.224,0.846)	0.014
**Safe**	16.7%	41	83.3	205	1	
**Reach of Animal**						
**Yes**	19.5%	26	80.5%	107	0.975 (0.548, 1.736)	0.933
**No**	19.2%	32	80.8%	135	1	

### Sanitation and hygiene factors associated with the lactulose to mannitol ratio

Regarding the association between environmental sanitation and an elevation of the lactulose to mannitol ratio (L:M > 0.15) in children, 14.3% (n = 23) of children from households with public use of latrines possibly had intestinal permeability or environmental enteropathy. Children from households with unsafe fecal disposal, open defecation, and fecal-smelling toilets 22.5% (n = 41), 18.9% (n = 47), and 14.2% (n = 27) had elevated lactulose-mannitol ratios, indicative of possible environmental enteropathy. Thirty-four (20.5%) of the children who had an elevated lactulose to mannitol ratio (>0.15) were from households that disposed of their waste improperly. On the bivariate analysis, households with their latrines open to the public (P = 0.018), fecal smell around the toilet (P = 0.001), flies and insects around the toilet (P = 0.004), showed a significant association with an increased lactulose mannitol ratio (indicative of possible environmental enteropathy). Households with unsafe disposal of child feces (P = 0.084), open defecation (p = 0.658), and improper solid and liquid waste management (p = 0.611), respectively, did not show a significant association with an increased lactulose to mannitol ratio (L:M > 0.15). Concerning the association between hygiene factors and an elevated lactulose to mannitol ratio, 12.8% (n = 24) and 22.1% (n = 52) of the children with an increased lactulose to mannitol ratio were from households that practiced hand washing less than the three critical times for hand washing and did not use any washing agents, respectively. The variables indicating that households practiced less than three critical times of hand washing and no agents were used during hand washing showed a significant association (p = 0.0001) and (p = 0.024) with an elevated lactulose to mannitol ratio (possibility of environmental enteropathy) in the bivariate analysis (Table 4).

**Table 4:** Association of sanitation and hygiene practice with lactulose to mannitol ratio of under-five children in slum areas of Jimma Town, Ethiopia; (N = 300).

	L:M> 0.15		L:M ≤0.15		Crude Odds Ratio (95% CI)	P-value
	Percent	Number	Percent	Number		
**Public use of latrine**						
**Yes**	14.3%	23	85.7%	138	2.019 (1.126,3.622)	0.018
**No**	25.2%	35	74.8%	104	1	
**Disposal of child feces**						
**Safe**	14.4%	17	81.6%	101	1.728(0.929,3.213)	0.084
**Unsafe**	22.5%	41	77.7%	141	1	
**Open defecation**						
**Yes**	18.9%	47	81.1%	202	1.182(0.565,2.475)	
**No**	21.6%	11	78.4%	40	1	0.658
**House hold solid waste disposal**						
**Proper**	18.1%	23	81.9%	104	1	
**Improper**	20.5%	34	79.5%	132	1.165(0.647, 2.097)	0.611
**Fecal smell**						
**Yes**	14.2%	27	85.8%	163	2.712(1.472,4.996)	0.001
**No**	28.2%	31	71.8%	79	1	
**Flies and insects at toilet Area**						
**Yes**	11.9%	18	81.1%	133	2.369(1.324,4.238)	
**No**	26.8%	40	73.2%	109	1	0.004
**Waste water disposal**						
**Proper**	16.9%	12	83.1%	59	1	
**Improper**	20.1%	46	79.9%	183	1.236(0.614, 2.488)	0.553
**Practice 3/less times of critical hand washing**						
**Yes**	12.8%	24	87.2%	163	2.923 (1.624, 5.260)	
**No**	30.1%	34	69.9%	79	1	0.001
**Agents used during hand washing**						
**Yes**	9.2%	6	90.8%	59	1	
**No**	22.1%	52	77.9%	183	2.794(1.142, 6.835)	0.024

A multivariable backward step-wise regression model was fitted to adjust for potential confounding. To rule out excess variables and unstable estimates in the final model, only variables that reached a p-value of less than 0.25 in the bivariate analysis were included in the multivariate analysis. After adjustment for potential confounders, elevated lactulose to mannitol ratio /possibly environmental enteropathy was found to be significantly associated the variables drinking water from unimproved water sources (AOR 3.741; 95%CI: 0.914–15.310,p = 0.048),water from unsafe coverage of water storage (AOR 0.363; 95%CI: 0.169–0.777, P = 0.009), public latrine utilization (AOR 0.139 95%CI: 0.024–0.816, P = 0.029),and hand washing less than 3 critical time of hand washing practices (AOR 4.369;95%CI: 1.411–13.524,P = 0.011) were significantly associated with an increased in lactulose mannitol ratio(indicative of possible intestinal permeability/environmental enteropathy). The variables Fecal smell at the toilet area (AOR = 1.633; 95% CI: 0.403–6.625), flies and insects at the toilet area (AOR = 2.635; 95% CI: 0.614–11.297), and no agents used during hand washing (AOR 2.345; 95% CI: 0.816–6.741) did not show a significant association, but they are more likely to have an elevated lactulose to mannitol ratio. In this finding, unsafe disposal of child faces (AOR = 0.329; 95% CI: 0.085–1.266) was less likely to have an elevated lactulose to mannitol ratio (Table 5).

**Table 5:** Shows bivariate and multivariable logistic regression models predicting the likelihood of lactulose mannitol ratio in slum areas of Jimma Town; (N = 300).

Variables		COR(95%CI)	P	AOR(95%CI)	P
**Water source**	Unimproved	3.262(1.808–5.888)	<0.0001	3.741(.914,15.310)	0.048
	Improved			1	
**General water storage practice**	Unsafe	0.597(0.312,1.140)	0.118	.602(.283,1.280)	.187
	Safe			1	
**House hold Water treatment**	Don’t use	599(.262, 1.370)	.225	.944(.315,2.828)	.918
	Use			1	
**Status of water storage coverage**	Unsafe	0.435(0.224,0.846)	0.014	.363(.169,.777)	.009
	Safe			1	
**Public use of latrine**	Yes	2.019(1.126,3.622)	0.018	.139(.024,.816)	.029
	no			1	
**Disposal of child feces**	Unsafe.	1.728(0.929,3.213)	0.084	.329(.085,1.266)	.106
	Safe			1	
**Fecal smell at the toilet area**	Yes	2.712(1.472,4.996)	0.001	. 1.633(.403,6.625)	0.493
	no			1	
**Flies and insects at the toilet area**	Yes	2.369(1.324,4.238)	0.004	2.635(.614,11.297)	0.192
	no			1	
**Practice 3 or less time of critical hand washing**	no.	2.923(1.624,5.260)	P = 0.001	4.369(1.411,13.524)	0.011
	Yes			1	
**washing agent used during hand washing**	no.	2.794(1.142–6.835)	0.024	2.345(.816,6.741)	0.114
	Yes			1	

## Discussion

The present study determined the prevalence and association of an elevated Lactulose-to-Mannitol Ratio (possibly indicative of environmental enteropathy) with water supply, sanitation, and hygiene. The prevalence of environmental enteropathy in the study area was found to be 19.3% (19.3%; 95% CI: 14.8, 23.7). The prevalence of an elevated lactulose to mannitol ratio (possibly due to environmental enteropathy) is high in children with an age group of 1–3, children from the age group of caretakers >35, family members ≥ 5, and housewives. Care givers with no education or primary education, and care givers with less income, have been observed to have a greater lactulose to mannitol ratio (>0.15) than their references. Although several risk factors were significantly associated with an increased Lactulose to Mannitol ratio (possibility of environmental enteropathy) in the bivariate analysis, some considerable factors that could predispose children were identified after adjusting for confounders. Variables including drinking water from unimproved water sources, water from unsafe cover storage, public latrine utilization, and hand washing less than 3 critical times of hand washing practice were significantly associated with an increase in the lactulose to mannitol ratio (>0.15).

The findings of this study revealed that the possibility of an elevated lactulose to mannitol ratio (indicative of environmental enteropathy) is high in children under the age of 1-3. The possible justification for lower age groups might be the low level of immunity in younger under-five children, the high risk of eating contaminated foods and drinks, the crawling starting times of under-five children, the high contamination rate of children’s hands; and low sanitation practices. This is similar to the study that justified that infants and young children in low income countries frequently come into contact with animal or human feces and contaminated soil while crawling and playing outdoors [23]. This is also justified by the study that indicate, the primary cause for EED was fecal ingestion due to living in poor water, sanitation and hygiene conditions [24].

According to this study, children from families with more than five members had an increased lactulose to mannitol ratio >0.15 (indicative of possible environmental enteropathy). This can be justified by the fact that when the number of children in the household increases, the quality of care and attention from parents decreases as mothers or caregivers become incapable of caring for children, so it is expected that children could be more vulnerable to contamination [14, 22]. The findings of this study indicated that children of housewives with no education and those with primary education were observed to have a higher level of lactulose to mannitol ratio than their references. This might be due to a lack of awareness of how to prevent exposure to unsafe sanitation and hygiene practices and a lack of awareness of the consequences of exposing children to poor sanitation [13]. Children from families with less income have been observed to develop higher levels of environmental enteropathy than their counterparts. As indicated by different studies, the possible explanation for this is, good income is related to better hygiene, sanitation, and frequent use of health services [14, 16, 22, 25]. Even though a study indicated that there is no significant associations between water source and either an elevated lactulose to mannitol ratio or stunting risk [20], According to this study, poor water, sanitation, and hygiene were some of the factors contributing to an increase in the lactulose to mannitol ratio. This was consistent with the hypothesis that unsanitary environmental conditions can lead to environmental enteropathy.

Despite the fact that environmental enteropathy can be caused by a variety of factors, it is frequent in places with poor water quality, sanitation, and hygiene, according to various studies. Unsafe water, sanitation, and hygiene practices were also mentioned as contributing factors to the development of environmental enteric dysfunction [1–21]. The findings of this study revealed that water from unimproved water sources, from unsafe water storage practices, and from unsafe cover of water storage showed a significant association with an increase in the lactulose to mannitol ratio. This is justified by a study that indicated that clean households with good quality water and sanitation had an inverse relationship with the lactulose to mannitol ratio, resulting in better intestinal health than children who live in contaminated households [26]. Unimproved water sources are prone to contamination with human excreta. The practice of water storage and the safety of its coverage also have an impact on the transmission of pathogenic organisms [26]. Water from unsafe storage with unsafe coverage is directly exposed to pathogenic organisms that lead to the ingestion of enteric pathogens with the water [2]. Drinking unsafe water and untreated excreta contaminate ground water and surface water used for drinking, irrigation, bathing, and household purposes and impairs health through illnesses such as diarrhea [27]. The result of the study done on the association of unsafe drinking water with environmental enteric dysfunction in rural southwest Uganda indicated that contaminated household drinking water may be an important contributing factor to the high burden of both EED and stunting in low- and middle-income countries with poor WASH conditions [20]. Environmental enteropathy is a subclinical condition generally thought to be caused by constant fecal-oral contamination [3]. Chronic exposure to fecal pathogens is hypothesized to cause inflammation and resultant structural changes in the small bowel, which ultimately result in functional changes [25].

The fecal-oral route, as described in the F Diagram (fluids, fingers, fields, flies, and food), is thought to be an important pathway for enteric infections [27]. Despite the fact that this study’s findings indicated that feces disposal was not associated with an increase in the lactulose to mannitol ratio, there was a significant association with public latrine use. This study also indicated that fecal odors and flies around the toilet were more likely to cause an increase in the lactulose to mannitol ratio. Safe disposal of human excreta creates the first barrier to excreta-related disease, helping to reduce transmission through direct and indirect routes. Safe excreta disposal is therefore a major priority, and in most disaster situations it should be addressed with as much speed and effort as the provision of a safe water supply. This is also consistent with the study that supports the direct relationship between the number of people using the toilet and As the number of people using the toilet increases, there is a chance of decreasing the cleanliness of the toilet, leakage of fecal material to the surface, and exposure to flies and other insects for transmission of disease-causing organisms through the fecal-oral route [27]. This is also justified by a review of studies that indicated that direct ingestion of fecaly contaminated soil and/or animal feces is a critical pathway for exposure to fecal pathogens by infants and young children [13].

It also indicated that infants and young children ingest dirt and feces through mouthing soiled fingers, play objects, and household items, as well as through exploratory ingestion of contaminated soil and/or poultry feces. Soil ingestion among infants and young children is associated with an increased risk of diarrhea, elevated markers of EED, and stunting [13].

According to the findings of this study, most caregivers wash their hands less than three times during the five critical times. It also showed a significant association with an increase in the ratio of lactulose to mannitol (indicative of environmental enteropathy), This is consistent with studies that indicated, practice of hand-washing at five critical times is an important factor in preventing the ingestion of pathogenic organisms indirectly from the contaminated hands of caregivers [24, 28].

The findings of this study also showed a significant association between no usage of hand washing agents and an increased lactulose to mannitol ratio (>0.15). This study has wider practical implications. The link between environmental enteropathy and child stunting has been reported in several studies [2, 3, 5, 7, 8, 10–12, 16–21]. Given the fact that there is a very high toll of stunting in Ethiopia, the findings call for strengthening interventions that prevent the exposure of children to unsafe environments to curb the problem of environmental enteropathy and associated stunting.

Even though it’s been done in lots of countries, the lactulose-mannitol test has never been done in Ethiopia as a biomarker for gut leakiness (permeability), which was used in this study. Performing the test with other supportive diagnostic measures will give an objective, indicator-based baseline for further study on environmental enteropathy and problems associated with it.

### Limitation

The findings of this study found that there is an association between water sanitation and hygiene factors and an elevated lactulose to mannitol ratio, which is indicative of the possibility of environmental enteropathy. Although environmental enteric dysfunction is due to different causes, due to a lack of diagnostic facilities, we did not perform other supportive tests, especially an intestinal biopsy, which is the gold standard to diagnose EED, to identify a specific cause (in addition to a water sanitation and hygiene problem) for an elevation in the lactulose to mannitol ratio (>0.15). As the study was done in slum areas with basic water sanitation and hygiene problems, It was better to keep variables as levels of services rather than taking them as binary (yes/no) to prevent losing some sensitivity in the analysis.

## Conclusion

The findings showed that poor water sanitation and hygiene practices were factors that contributed to an increase in the lactulose-mannitol ratio (possibly indicative of environmental enteropathy). Despite the fact that environmental enteropathy has different causes and that having diagnostic facilities to identify children with this problem and their specific causes is a challenge in the study setting, other supportive diagnostic measures were not performed. Based on the findings of this study, we proposed an intervention plan by recommending responsible sectors, such as the WASH (water, sanitation, and hygiene) sector, focus on areas with a shortage of WASH facilities; the health sectors, raise society’s awareness of how to prevent the occurrence of problems related to poor WASH; and focus on diagnostic measures of environmental enteropathy. As there is a research gap on the consequences of environmental enteropathy in terms of growth failure, oral vaccine failure, cognitive development, and the general health condition of children, filling the gap is our future plan.
